# Improving the aseptic transfer procedures in hospital pharmacies. Part B: evaluation of disinfection methods for materials with a non-sterile surface

**DOI:** 10.1136/ejhpharm-2018-001673

**Published:** 2019-08-24

**Authors:** Frits A Boom, Paul P H Le Brun, Stefan Boehringer, Jos G W Kosterink, Daan Touw

**Affiliations:** 1 Zaans Medical Center, Zaandam, The Netherlands; 2 Department of Clinical Pharmacy & Toxicology, Leiden University Medical Center, Leiden, The Netherlands; 3 Medical Statistics and Bioinformatics, Leiden University Medical Center, Leiden, The Netherlands; 4 Department of Clinical Pharmacy and Pharmacology, University of Groningen, University Medical Center Groningen, Groningen, The Netherlands

**Keywords:** aseptic handling, colony-forming unit, disinfection, spore-forming bacteria, surface bioburden

## Abstract

**Objectives:**

To improve the disinfection methods for materials with a non-sterile surface to be used in aseptic handling.

**Methods:**

The surface bioburden on ampoules (A) and injection vials (IV) is determined by contact plates and total immersion. The occurrence of spore-forming bacteria is determined by strain colouring and matrix-assisted laser desorption ionisation-time of flight mass spectrometry. The disinfection procedures of non-sterile materials in 10 hospital pharmacies are judged by observing.

**Results:**

After wiping according to local disinfection methods, the mean surface bioburden determined by contact plates in 10 hospital pharmacies is 0.36 (plastic A), 0.50 (glass A) and 0.29 colony-forming unit (cfu) (IV). The observers found great differences in accuracy of wiping and degree of wetting the sterile gauzes.

After improved wiping with commercially available alcohol impregnated sterile wipes and a two-towel technique (one-step TT disinfection), the mean surface bioburden determined by contact plates is 0.03 (plastic A), 0.2 (glass A) and 0.13 cfu (IV). Further improvement can be reached by submerging A and IV in ethanol 70% followed by improved wiping (two-step TT disinfection), but still micro-organisms will remain (mean surface bioburden determined by total immersion is 0 (plastic A) and 0.3 cfu (IV); glass A not determined). Two-step TT disinfection is more labour intensive. Spilling of alcohol is another disadvantage. However, we presume one-step TT disinfection is effective enough in daily practice. Routine surface bioburden determinations have to prove this.

The effectiveness of the combination of spray and wipe is not examined because we observed a quick disappearance of alcohols from vertical as well as horizontal surfaces, which shortens the contact time to far below the advised 2 min.

Spore-forming bacteria disappear as quickly as other micro-organisms during disinfection by alcohols.

**Conclusion:**

Local disinfection procedures can be improved. Complete removal of micro-organisms from materials with a non-sterile surface, even after two-step TT disinfection, is impossible. Routine surface bioburden determinations have to prove if one-step TT disinfection is effective enough.

## Introduction

Materials with a non-sterile surface, like ampoules, vials and bottles, can drag micro-organisms into a laminar airflow cabinet (LAF), a safety cabinet (SC) or an isolator (I) and may contaminate the hands of the operator. Contaminated hands as well as contaminated materials risk contaminating the product. Materials with a non-sterile surface must therefore be disinfected before being transferred into LAF/SC/I. The result of this disinfection depends on the surface bioburden, the disinfectant used and the disinfection method.

The surface bioburden before disinfection can be low if enough precautions are taken. This will be worked out further in part C of this series of articles. The preferable method for the determination of the surface bioburden has been discussed in part A.^1^

The disinfectant most widely used in the Netherlands and in most other European countries is based on alcohol (ethanol or isopropyl alcohol 70%). After application, it will dry quickly without leaving any residues. However, alcohols are not effective against bacterial spores. Whether or not this is a relevant shortcoming has never been investigated to our knowledge.

A combination of spraying and wiping is the advised disinfection method.^2–4^ Wiping is a combination of inactivation by a disinfectant and mechanical removal of micro-organisms. By spraying the disinfectant can penetrate into difficult-to-reach surfaces. However, the impact of alcohol aerosols on the operators restricts the use of spraying.^5^ Therefore, wiping only is the preferred method of disinfection in the Netherlands, but its efficacy has not been examined.

In pharmaceutical industries, submersion of materials into a disinfectant is a well-known disinfection method for materials with a non-sterile surface, like ampoules and vials. In this way, the contact-time can be guaranteed much better and all surfaces, including spots that are difficult to reach, will be in contact with the disinfectant.

In this article, we try to answer the following research questions:

- Which disinfection method is the most practical to reach a low surface bioburden on materials with a non-sterile surface?
- Is the use of a sporicide, as part of the disinfection procedure, necessary?

## Materials and methods

### Surface bioburden on materials after disinfection in 10 hospital pharmacies

1. *Disinfection and bioburden determination*: in all participating hospital pharmacies 10 samples of 10 mL plastic ampoules, 10 mL glass ampoules, 10 mL glass injection vials and 100 mL glass infusion vials were used for this investigation. The sampled ampoules and vials are products used in parenteral nutrition preparation. Samples were disinfected by wiping with sterile gauzes wetted with ethanol 70% according to the local disinfection method (one-step LP disinfection) and transferred into LAF or SC after disinfection. Surface bioburdens were determined by contact plates as described in part A.^1^
2. *Judging of the local disinfection procedure*: two experienced operators observed one-step LP disinfection of non-sterile materials in the 10 hospital pharmacies.

### Development of two standardised disinfection methods

We developed two disinfection methods with a precise description of the disinfection procedure.

**Figure 1:**
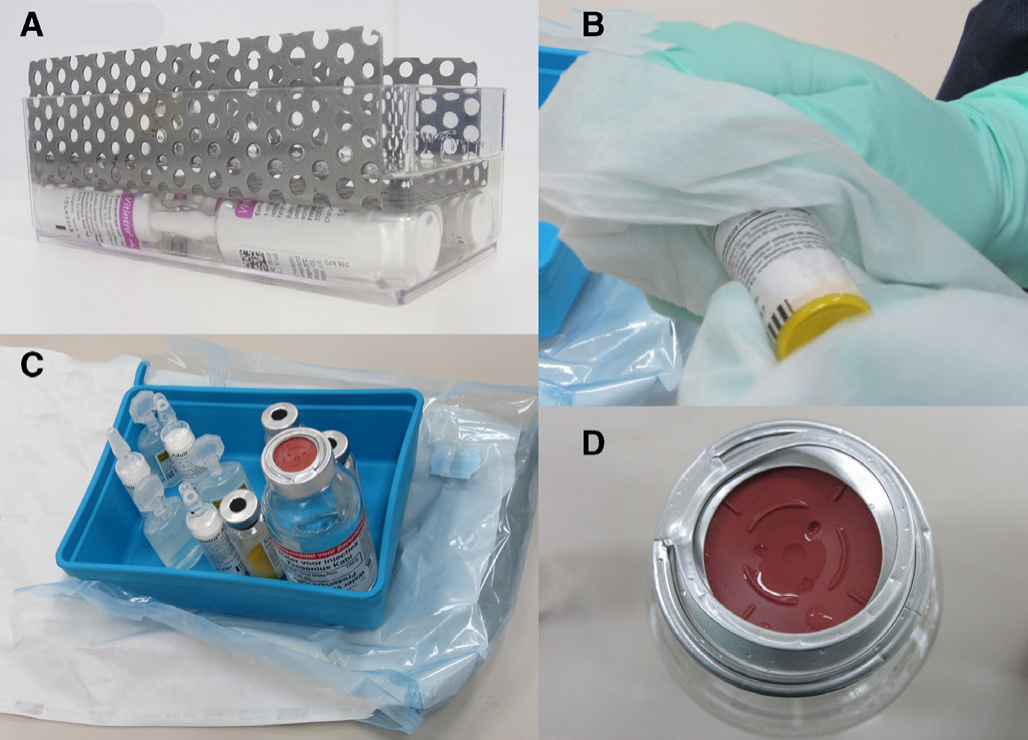
Steps of the disinfection procedure of ampoules and vials. (A) Submersion of ampoules in alcohol 70%. (B) Two-towel wiping technique. (C) Filled sterile tray on a sterile surface. (D) droplets on top of a vial just after spraying with an alcohol.

1. *One-step two-towel disinfection method (one-step TT disinfection)*: this method was the same as the two-step disinfection method (see 2), but without submerging and putting afterwards vials and ampoules in a flush disinfected tray.
2. *Two-step disinfection method (two-step TT disinfection)*: glass ampoules (Supliven 10 mL, Vitintra adult 10 mL, both Fresnius-Kabi) and injection vials (Soluvit N, Fresenius-Kabi) were collected straight from their original boxes and transferred into a clean and disinfected flush tray. Plastic flip-off caps were removed from vials. Vials and ampoules (samples) were submerged in ethanol 70% for at least 2 min using a flush transparent plastic tray and a metal tool to support submersion (figure 1A) and put afterwards in a flush disinfected tray. Samples were wiped thoroughly by the two-towel technique, which means wiping with two impregnated sterile polypropylene wipes (isopropyl alcohol 85% and demineralised water 15%, 227×279 mm, Prosat, Contec) (figure 1B). Finally, the samples were placed in a sterile tray standing on a sterile surface (figure 1C).

### Comparing the effectiveness of the two standardised disinfection methods

The effectiveness of the one-step TT as well as the two-step TT disinfection has been tested in an experimental setting.

1. *Sampling:*
  - *Sampling before disinfection*: glass ampoules (Supliven 10 mL, Vitintra adult 10 mL, both Fresnius-Kabi) and injection vials (Soluvit N, Fresenius-Kabi) were taken aseptically from the original boxes and transferred into a LAF cabinet.
  - *Sampling after one-step TT and two-step TT disinfection*: the sterile trays with samples (figure 1 C) were placed into a LAF cabinet.
2. *Bioburden determination*: surface bioburdens were determined by contact plates and total immersion as described in part A of this series of articles.^1^ We have restricted the experiments with total immersion to plastic ampoules and injection vials because of the costs.

### Statistics

The results were statistically analysed by negative binominal regressions to account for overdispersion in the data (ie, a mean/variance relationship).^6^ P values were calculated using a Wald test.^7^ A p value <0.05 is considered to be statistically significant. P values were computed for the comparison of disinfection methods. Within a single type of container, no covariate is added to the regression, when combining all containers in a single analysis, container type is used as a covariate.

### Identification of micro-organisms

Micro-organisms found on the materials by contact plates (see above ‘bioburden determination’) were identified by microscopic examination and strain colouring and divided in Gram-positive cocci and rods, Gram-negative cocci and rods, moulds and yeasts. Gram-positive rods were further identified in non-spore forming and spore forming by spore colouring or by matrix-assisted laser desorption ionisation-time of flight mass spectrometry (MALDI-TOF MS).^8^ Micro-organisms found by total immersion (see above ‘bioburden determination’) were divided in the same groups by MALDI-TOF MS only.

## Results

### Surface bioburden on materials after disinfection in 10 hospital pharmacies

**Figure 2:**
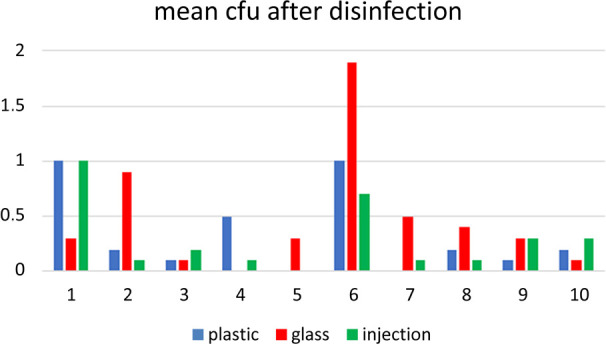
Surface bioburden expressed as mean cfu (n=10) on plastic and glass ampoules and on injection vials in 10 hospital pharmacies after one-step disinfection according to the local procedure. Used technique: contact plate. Horizontal axis: 1, 2, 3, etc are hospital 1, 2, 3, etc. Vertical axis: mean cfu/sample. cfu, colony-forming unit.

Figure 2 shows the surface bioburden on materials in 10 hospital pharmacies after one-step LP disinfection.

During the observations, great differences in accuracy of wiping and degree of wetting the sterile gauzes were found. This is not surprising because disinfection of materials was not fully described in the standard operating procedures (SOP) in all participating hospital pharmacies.

### Comparing the effectiveness of different disinfection methods

1. One-step LP and one-step TT disinfection:

Table 1 shows the mean surface bioburdens after one-step LP disinfection (results figure 2) and after one-step TT disinfection. Bioburdens are determined by contact plates. The results show less remaining cfu after one-step TT compared with one-step LP disinfection for all three types of containers.

**Table 1:** Surface bioburden expressed as mean cfu per ampoule or vial in 10 hospital pharmacies after one-step LP disinfection and in an experimental setting after one-step TT disinfection

	Plastic ampoules		Glass ampoules		Injection vials		All kinds of containers	
	Mean cfu*	SD	Mean cfu*	SD	Mean cfu*	SD	Mean cfu*	SD
one-step LP	0.36 (n=100)	1.15	0.50 (n=90)	0.94	0.29 (n=100)	1.07	0.38 (n=290)	1.06
one-step TT	0.03 (n=30)	0.18	0.20 (n=30)	0.41	0.13 (n=30)	0.43	0.12 (n=90)	0.36
p	0.029		0.073		0.241		0.002	

*cfu determined by contact plate.

cfu, colony-forming unit; n, number of samples examined; p, p value; one-step LP is reference group.

The comparison of one-step LP and one-step TT disinfection was analysed both per sample type (plastic, glass, injection) and in a combined analysis. The combined regression included sample type as a covariate. Two samples after one-step LP disinfection, one plastic ampoule and one injection vial, could not be counted (over 100 cfu). These measurements were set to 10 cfu. This leads to conservative p values as the difference between groups is diminished. When comparing single container types, the only significant difference was found for plastic ampoules. For these analyses, power was limited by small cfu values, combined with large SD. The overall comparison (all kinds of containers), looking for differences between any types of containers, is significant.

­

2. One-step TT and two-step TT disinfection:

Table 2 shows the mean surface bioburdens before disinfection, after one-step TT (the same results as used in table 1) and after two-step TT disinfection. Bioburdens are determined by contact plates as well as by total immersion.

**Table 2:** Surface bioburden expressed as mean cfu count per ampoule or vial before disinfection and after one-step TT and two-step TT disinfection

	Plastic ampoules				Glass ampoules		Injection vials			
	Contact plate		Total immersion		Contact plate		Contact plate		Total immersion	
	Mean cfu	SD	Mean cfu	SD	Mean cfu	SD	Mean cfu	SD	Mean cfu	SD
Before disinfection	0.67 (n=30)	1.64	2.45 (n=20)	2.50	0.93 (n=30)	1.82	0.57 (n=30)	1.04	7.35 (n=20)	17.26
After one-step TT	0.03 (n=30)	0.18	0.10 (n=20)	0.46	0.2 (n=30)	0.41	0.13 (n=30)	0.43	1.35 (n=20)	3.67
After two-step TT	0.03 (n=30)	0.18	0.00* (n=20)	0.00	*0.00* (n=30)	0.00	0.03 (n=30)	0.8	0.30 (n=20)	0.56
p_1_	0.010		<0.001		0.007		0.022		0.003	
p_2_	0.010		<0.001		0.002		0.008		<0.001	
p_3_	1.000		0.679		0.218		0.304		0.423	

*For the Wald test the mean (x̅) of zero cfu was replaced by a pseudo-count of 1 (the lowest possible contamination: x̅=0.05 in 20 samples and x̅=0.03 in 30 samples).

cfu, colony-forming unit; n, number of samples examined; p_1_, p value; one-step TT disinfection compared with before disinfection;p_2_, p value; two-step TT disinfection compared with before disinfection; p_3_, p value; two-step TT disinfection compared with one-step TT disinfection.

Count of cfu was regressed against disinfection status (before disinfection, after one-step TT, after two-step TT). Regressions were performed twice, once with ‘before disinfection’ and once with ‘after one-step TT disinfection’ as reference group. P values (p_1_, p_2_ and p_3_), corresponding to the different comparisons, are explained in the footnote of table 2. To be able to analyse samples for which zero cfu were observed, this value was replaced by a pseudo-count of 1. This leads to conservative p-values as the difference between groups is reduced.

Both, one-step and two-step TT showed significantly smaller cfu counts for all container types (p_1_ and p_2_). The cfu counts after two-step TT were lower than after one-step TT for all container types but the differences were not statistically significant. For this comparison, statistical power was low due to small cfu values and large SD.

### Identification of micro-organisms found on materials before and after disinfection

The results of the identification of the micro-organisms found on the materials as displayed in table 2 are summarised in table 3.

**Table 3:** Identification of cfu found on materials by contact plate and total immersion

		G+ coc	G− coc	G+ rod	G+ rod sp	G− rod	Mould	Yeast	All cfu
Contact plate	Before disinfection	31	0	11	19	1	3	0	65
	After one-step TT	0	0	6	3	2	0	0	11
	After two-step TT	0	0	1	1	0	0	0	2
Total immersion	Before disinfection	50	0	1	135	0	1	9	196
	After one-step TT	0	0	0	28	1	0	0	29
	After two-step TT	2	0	1	2	1	0	0	6

G+ coc, Gram-positive coccus; G− coc, Gram-negative coccus; G+ rod, Gram-positive rod; G+ rod sp, Gram-positive rod spore forming; G− rod, Gram-negative rod.

To get a better insight into the disappearance of the spore-forming bacteria by disinfection, the percentage of remaining spore-forming bacteria and all micro-organisms after one-step TT and after two-step TT disinfection were calculated. Results are summarised in table 4.

**Table 4:** Remaining Gram-positive rods spore-forming bacteria (G+ rod sp) and all micro-organisms (all cfu) after one-step TT and after two-step TT disinfection (percentage remaining between brackets)

	Contact plate		Total immersion	
	G+ rod sp	All cfu	G+ rod sp	All cfu
Before disinfection	19 (100%)	65 (100%)	135 (100%)	196 (100%)
After one-step TT disinfection	3 (16%)	11 (17%)	28 (21%)	29 (15%)
After two-step TT disinfection	1 (5%)	2 (3%)	2 (1%)	6 (3%)

## Discussion

### Surface bioburdens after disinfection in 10 hospital pharmacies

Considerable differences in remaining cfu on the materials in the 10 hospital pharmacies are found after one-step LP disinfection (figure 2). The accuracy of wiping and the degree of wetting of the sterile gauzes are the most plausible explanations for this. The lack of a more precise description of the disinfection procedure of non-sterile materials in the reviewed SOPs can be considered as a shortcoming.

To eliminate the effect of insufficient wetting, the use of commercially available alcohol impregnated sterile wipes is advised.^9^ To reach the ampoule or vial surface better by the wipes, the two-towel wiping technique can be helpful (figure 1B). Both are used in the two standardised disinfection methods (one-step and two-step TT).

A precise description in the SOP of the way surface disinfection has to be performed is uncommon in Dutch hospital pharmacies. Therefore, results as we found in the 10 hospital pharmacies after one-step LP disinfection can also be expected in other hospital pharmacies in the Netherlands.

### Improving the disinfection procedure

As mentioned earlier, a combination of spray and wipe is the generally advised disinfection method.^2–4^ Studies to show the superiority of this combined method over wiping have only been done with small numbers of samples and are executed using artificially contaminated surfaces, of which the contamination levels are much higher than those found on non-sterile materials in practice. Moreover, results from previous studies were not always consistent.^10–12^

There are additional reasons to doubt the efficacy of spray and wipe. First, the penetration of alcohol into difficult-to-reach surfaces (eg, under the crimp cap of vial seals) is not guaranteed and, second, after spraying the disinfectant film disappears quickly by dripping down from vertical surfaces and droplets forming on horizontal surfaces, as is shown in figure 1D. Therefore, in practice the contact time after spraying is too short to be effective.

In the pharmaceutical industry the submersion of materials in a disinfectant is a well-known method for materials to be used in an EU GMP Annex 1 grade A-environments.^13^ We therefore added a submersion step to one-step TT disinfection. When comparing both, we used the contact plate method and total immersion. The last is more sensitive and thus necessary to solve questions about the effectiveness of a disinfection method.^1^

The results after two-step TT are better than after one-step TT but the differences are not statistically significant (table 2). The question remains which of both methods has to be advised. The first step in two-step TT, submersion, makes disinfection of materials less operator dependent. In combination with the different trays (see section ‘Materials and methods’), it is better organised, so line clearance is easier to execute. Spilling of alcohol on the workbench however cannot be avoided and obviously two-step TT disinfection takes more time in comparison with one-step TT disinfection.

One-step TT disinfection, as described in this article, also uses different trays with the accompanying advantage of a better line clearance. This method shows good results for glass and plastic ampoules, but micro-organisms on difficult-to-reach surfaces, for example, injection vials, cannot be reached. The question is whether these hidden micro-organisms are a risk for the contamination of products prepared in LAF/SC/I. If they are hidden from contact plates, they also are hidden from the fingers of the operator.

In spite of the moderate disinfection procedure of non-sterile materials in Dutch hospital pharmacies (figure 2), the results of process validation of aseptic handling with a broth solution are in general good (<1 contaminated sample in a 1000 samples).^14^ Like in ‘standard’ environmental and surface microbial monitoring, a correlation between monitoring results and results from process validation is hard to find. However, disinfected non-sterile materials can drag micro-organisms inside LAF/SC/I, which is a risk of non-sterility. According to risk assessment principles, we have to keep this risk as low as possible, which means an effective disinfection procedure, controlled regularly. Therefore, further studies in actual practice are required to demonstrate whether one-step TT disinfection is effective enough.

### Spore-forming bacteria as part of the surface bioburden

Because of a number of patient deaths related to *Bacillus cereus* found in aseptically prepared products in the UK, wiping with an alcoholic wipe in the first stage of the two-stage transfer disinfection process in the UK, was replaced by wiping with a sporicidal disinfectant.^15–17^ Data to prove the superiority of this change however are absent to our knowledge. *B. cereus* is a spore-forming bacterium (Gram-positive rod spore forming). This bacterium can form spores under good as well as bad growing conditions. Alcohol-based disinfectants are effective against spore-forming bacteria if they are in the vegetative phase but are ineffective if they are in the spore phase.

We found a considerable number of spore-forming bacteria on ampoules and injection vials before disinfection (table 3). The reduction of these bacteria after one-step and two-step disinfection is nearly the same as the reduction of all kinds of micro-organisms (table 4). Therefore, most of the spore-forming bacteria must be in the vegetative phase. Based on these findings, we doubt the benefits of the addition of a sporicidal disinfectant. Besides there are more reasons to be doubtful. First, studies executed with bacterial spores show mechanical removal of spores by wiping (wiping is a part of one-step and two-step TT disinfection).^11 18^ Second, complete removal of viable micro-organisms from ampoules and vials after disinfection is impossible (table 2). Third, the effectivity against bacterial spores for the most used sporicidal disinfectant (6% hydrogen peroxide) is low.^19^ Company tests of Klercide Sporicidal Low Residue Peroxide (6% hydrogen peroxide) states that a contact time of 15 min is needed for sporicidal action.^17^ In practice, such a long time will not be applied during disinfection of materials, which reduces the sporicidal action substantially. We therefore decided to continue the disinfection of materials with an alcohol-based disinfectant only.

## Conclusion

Disinfection of non-sterile materials in Dutch hospital pharmacies needs to be improved by a more precise description of the disinfection procedure in the local SOP and more accuracy of wiping.

Complete removal of micro-organisms from materials with a non-sterile surface, even after two-step TT disinfection is impossible. Disinfection of materials by thorough wiping only with well impregnated sterile wipes (one-step TT disinfection) seems to be effective enough. However, microbiological monitoring of surfaces after implementation of one-step TT disinfection as a routine procedure has to prove this.

Spore-forming bacteria disappear as quickly as other micro-organisms during disinfection by alcohols. Therefore, weighing the pros and cons, we continued disinfection of materials with an alcohol-based disinfectant only.

**What this paper adds**

**What is already known on this subject**

- A combination of spray and wipe is the generally advised surface disinfection method for ampoules and vials.
- The added value of spraying in combination with wiping is doubtful.
- The impact of alcohol aerosols on the operators is a great disadvantage of spraying.

**What this study adds**

- Complete removal of micro-organisms from ampoules and vials by surface disinfection is impossible.
- Improved wiping with well impregnated wipes seems to be effective enough in daily practice. However, routine surface bioburden determinations have to prove this.
- To support a good disinfection technique, a precise description in the standard operating procedures is important.

## Data Availability

Data are available on reasonable request.
